# MNEMONIC: MetageNomic Experiment Mining to create an OTU Network of Inhabitant Correlations

**DOI:** 10.1186/s12859-019-2623-x

**Published:** 2019-03-14

**Authors:** Aleksandra I. Perz, Cory B. Giles, Chase A. Brown, Hunter Porter, Xiavan Roopnarinesingh, Jonathan D. Wren

**Affiliations:** 1 Arthritis and Clinical Immunology Program, Division of Genomics and Data Sciences, Oklahoma Medical Research Foundation, Oklahoma City, OK 73104-5005 USA; 2 Department of Biochemistry and Molecular Biology, University of Oklahoma Health Sciences Center, Oklahoma City, OK USA; 3 Oklahoma Center for Neuroscience, University of Oklahoma Health Sciences Center, Oklahoma City, OK USA; 4 Department of Geriatric Medicine, University of Oklahoma Health Sciences Center, Oklahoma City, OK USA

**Keywords:** Human microbiome, Differential abundance, Case-control shift, Meta-analysis

## Abstract

**Background:**

The number of publicly available metagenomic experiments in various environments has been rapidly growing, empowering the potential to identify similar shifts in species abundance between different experiments. This could be a potentially powerful way to interpret new experiments, by identifying common themes and causes behind changes in species abundance.

**Results:**

We propose a novel framework for comparing microbial shifts between conditions. Using data from one of the largest human metagenome projects to date, the American Gut Project (AGP), we obtain differential abundance vectors for microbes using experimental condition information provided with the AGP metadata, such as patient age, dietary habits, or health status. We show it can be used to identify similar and opposing shifts in microbial species, and infer putative interactions between microbes. Our results show that groups of shifts with similar effects on microbiome can be identified and that similar dietary interventions display similar microbial abundance shifts.

**Conclusions:**

Without comparison to prior data, it is difficult for experimentalists to know if their observed changes in species abundance have been observed by others, both in their conditions and in others they would never consider comparable. Yet, this can be a very important contextual factor in interpreting the significance of a shift. We’ve proposed and tested an algorithmic solution to this problem, which also allows for comparing the metagenomic signature shifts between conditions in the existing body of data.

**Electronic supplementary material:**

The online version of this article (10.1186/s12859-019-2623-x) contains supplementary material, which is available to authorized users.

## Introduction

Communities of microbial species have co-evolved within a number of microenvironments, both outside and inside of larger organisms. For example, humans do not produce all the enzymes necessary to metabolize the spectrum of nutrients they take in as food, and the gut environment, through some uncertain mechanism, permits microbes that metabolize nutrients for the host in exchange for non-essential products to effectively be permanent yet dynamic co-habitants. However, pathogenic microbes can also occupy niches in the human body and often come with unwanted consequences for the host. The link between pathogenic microbes and acute conditions (e.g., diarrhea) has long been known but, in part, current studies are exploring whether or not chronic conditions may be due to changes in microbial communities. By cataloging microbial composition in these environments, we hope to better understand what role they may play in an array of physiological processes and diseases.

Changes in relative microbial abundance, known as differential abundances (DA), are a common measure of microbial variability and a starting point for understanding how certain mutualistic or pathogenic species may contribute to vital functions or diseases. Thus, changes in the relative abundance of microbial species could either cause or correlate with certain diseases, either by removing symbiotic microbes or by introducing hostile/non-beneficial microbes. And although a metagenomic experiment can quantify the shift in species abundance, interpreting its potential relevance and significance relies in part upon putting the newly observed shift within the context of previously observed shifts.

Metagenomic studies can be motivated by several goals, including the discovery of novel microbial genes of interest [1, 2], validation of metabolic hypotheses [3–5], profiling of the relationship between microbial community composition and variation in environmental or geographic parameters [6, 7] and assessment and comparison of the global metabolic complement found in one or more habitats [8–12]. In particular, there has been a substantial increase in examining potential associations between microbial changes and either chronic or late-onset human disease [13].

However, in human gut microbiome studies it is often the case that the composition of the microflora varies greatly depending on variables that are not directly related to a studied disease or condition, such as geographical location or project. The public availability of metagenomic data provides a powerful opportunity to corroborate the significance of microbial changes by searching for similar changes, thus showing robustness. The conditions in which the changes observed could either help corroborate one’s observations (e.g., if the experiment was a similar experiments) or could raise interesting questions (e.g, if the experiment was very different, yet yielded similar results).

Meta-analytic approaches are useful to identify statistically significant changes, but are likely limited when it comes to understanding the biological significance of microbial changes [14]. Nonetheless, identifying similar and opposing shifts in species abundance accelerates both one’s confidence in the robustness of the results and biological interpretation of the changes.

### The gut microbiome

**Fig. 1:**
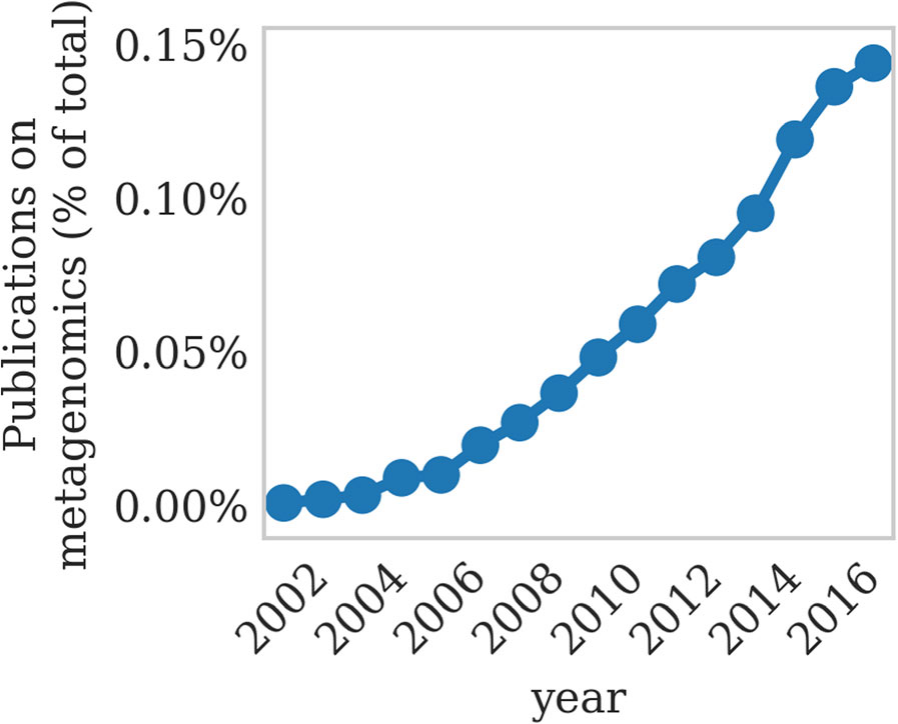
The percentage of publications in PubMed containing the word “metagenomics” per year is growing rapidly relative to the overall growth of PubMed

**Fig. 2:**
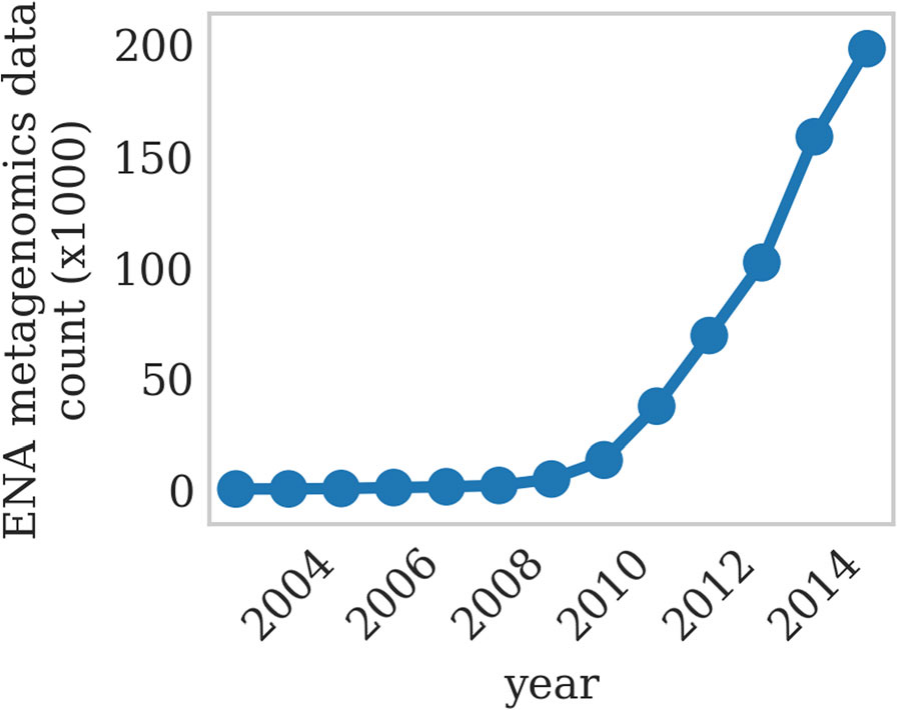
Growth of metagenomics data in ENA

Although the number of experiments that could be analyzed is growing rapidly (Figs. 1 and 2), because they lack standardized meta-data and annotations describing which sets belong to experimental groups and which to control groups, automatically determining this is still an open problem. However, the American Gut Project (AGP) [15] is one of the largest studies conducted to date and has a highly structured description of potential covariates, such as dietary preferences, so we chose the AGP subset for analysis.

The gut microbiome also has the advantage that it has been studied in a variety of contexts and has been shown to change in a range of diseases. Gastrointestinal tract and metabolic diseases have been studied particularly extensively, but the effect of gut microbes seems to be broader than that: it has been suggested that it might be implicated in autoimmune, mental, and cardiovascular diseases as well. Rheumatoid arthritis (RA) and inflammatory bowel disease (IBD) [16–18] diarrhea [19] as well as antibiotic treatment were shown to decrease the microbiota diversity. Further, obesity, metabolic syndrome, and type II diabetes [7, 11, 20–22], colitis [23, 24], colorectal cancer [25] have all been shown to be associated with changes in microbiome. There is some evidence that the microbiome might be implicated in autism spectrum disorders and cardiovascular disease, but the exact nature of this link has not been established ([26–28].

Factors that have been shown to affect the microbiome composition of the human gut include diet, geographical location, culture, and genetics [11, 29–36]. Specifically, there seems to be a great difference between the Western and plant-based diet, to the point of distinguishing human gut ‘enterotypes’ based mostly on prevalence of taxa that are associated with these diets. Consumption of salt can increase the prevalence of specific genes in the gut [37].

Changes in the gut microbiome also occur during late pregnancy [38]. Route of delivery affects the initial gut microbiome [39, 40], and the samples from infant gut cluster more readily with samples from human vagina than adult gut [41]. The largest changes are seen within the first three years of life [31, 41–45].

Aging has also been shown to change the gut microbiota, specifically to increase the number of ‘subdominant species’ [42].

Thus, a number of connections between microbiome changes in the gut and human health have been established. Part of the growing scientific interest in metagenomics studies is the therapeutic potential for intervention. If we can establish and understand links between species presence and/or relative abundance and human disease, there are a number of safe and inexpensive ways the microbiome could be altered in an attempt to alter the course of the disease. Potential clinical applications include supplementation of beneficial bacteria to supplement or help regulate host metabolism, metabolize molecules that might be problematic within the human digestive tract, and identifying microbes that might serve as natural competitors to harmful species.

### Metagenomics resources

Along with the rush to sequence microbial genomes within their environment, a number of bioinformatics resources were developed for storage, functional and taxonomic analysis, visualization, and retrieval of data. There are a number of repositories where users can store and browse metagenomic data: Community Cyberinfrastructure for Advanced Microbial Ecology Research and Analysis CAMERA (no longer operating, but the data is still available through iMicrobe) [46], Integrated Microbial Genomes and Metagenomes IMG/M ([47] Metagenomics-Rapid Annotations using Subsystems Technology MG-RAST ([48]), and EBI Metagenomics ([49]). They offer storage of sequencing data, as well as processing and basic functional and taxonomic analyses. Raw sequencing reads may be stored in databases like SRA or ENA. SEED and KEGG databases are often used as the reference for the functional components of the metagenome.

Taxonomic abundance change (i.e., differential abundance between conditions), can be analyzed with MEGAN, Dendroscope3, LEfSe [50], ANCOM [[51]. as well as a number of dedicated R packages (Phyloseq, metagenomeSeq,, and MaAsLin [52], BhGLM [53], as well as R packages primarily used for RNA-Seq data and adapted to microbiome analysis (DESeq2, edgeR [54], limma-voom [55] and web applications (Metastats); QIIME and MEGAN aim to integrate many analysis steps into a pipeline, which may also include functional analysis. QIIME is a widely-used and rich suite of tools for command-line analysis and visualization of sequencing metagenomics data that also integrates other tools [56]. Building on top of some of the previously mentioned tools, Nephele offers a web interface and the ability to compare the uploaded data to a data from a selected body site from Human Microbiome Project [12, 57]). A specific type of analysis may also be performed with eudysbiome - an R package whose flag concept is the dichotomous character of host-microbe interaction, or MetaMIS, which simulates the interactions between microbes in a group of samples across time.

While many of these packages can aid investigators in identifying species that change between two conditions of interest, they are focused on many pairwise comparisons and lacking in the ability to compare entire vectors of changes. By focusing on comparing these vectors of differential abundance, DA shifts, our tool empowers researchers to both build confidence in their results by comparing to similar experiments and gain novel insight into microbiome changes by comparing to other shifts associated with perturbations and pathologies.

## Mnemonic

We present a tool, MNEMONIC (MetageNomic Experiment Mining to create an OTU Network of Inhabitant Correlations), whose main goal is to calculate microbial shifts occurring in different conditions and then compare those shifts between the conditions, thereby assessing the similarity of conditions in terms of how the microbiome changes. It allows for the exploration of different shifts, as well as cross-referencing those changes with literature association data and microbial traits. One can also provide their own data and compare their shifts to the shifts that we observe in the AGP data. MNEMONIC is a python package and is publicly available for download at https://gitlab.com/wrenlab/mnemonic. It uses the publicly available data from the EBI Metagenomics portal by fetching microbial count data for the samples within the American Gut Project. To model the differential abundance between groups of samples it uses the R package edgeR. For other tasks, python was used.

One type of question that can be tackled with this approach is which taxonomic groups shift in the same or opposite direction as a response to a certain condition and whether there are other conditions that a similar change is seen in. Conceivably such shared shifts in microbiome may indicate that similar mechanisms are in play. For example a certain dietary habit might influence the host microbiome in a specific way, favoring certain species over other. A now highly-abundant species might influence the host’s health state eliciting or reducing an immune response from the host, or affecting the host via a product of microbial metabolism. Diseases may have a common cause in terms of the microbiome composition and function that, if evaluated, may allow a researcher to form hypotheses on whether a certain condition may be improved with a treatment that is commonly used for another.

Another example of a hypothesis that can be addressed is evaluating which taxonomic groups shift with - or opposite to - each other regularly and would allow to make statements about the interactions between the microbes themselves.

To the best of our knowledge, no other software has been described that is capable of comparing and visualizing shifts between the differential abundance vectors between conditions.

In addition to this novel functionality, MNEMONIC is also capable of bringing publicly available information as a context for a new study. For example, if a researcher plans a study on how the microbiome changes in diabetic people as compared to the non-diabetic people, MNEMONIC can provide a summarization of the results for this comparison from the AGP data. This is very useful for validating, as well as contrasting, the results of a new study.

## Methods and implementation

### Metagenomics data

There are two major approaches to obtaining metagenome data. One is amplification of bacterial 16S rRNA hypervariable regions, followed by amplicon sequencing and assigning the sequences to specific operational taxonomic units (OTUs). This approach can detect bacteria only. The hypervariable region may not be specific to a species, therefore sometimes only allows annotation to a higher taxonomic level. Gene presence in the sample cannot be obtained directly from the DNA, and can only be estimated based on the OTU presence.

Another common approach is whole genome shotgun sequencing. WGS provides information about the full DNA sequence in the sample, therefore allowing for a more accurate, as well as more sensitive [58]. species annotation. It can also identify sequences form kingdoms other than bacteria. Is however more expensive and requires a high coverage [59] and the analysis is computationally heavier. WGS also allows to directly map DNA sequences to a gene reference database for functional profiling.

### Data characterization

**Fig. 3:**
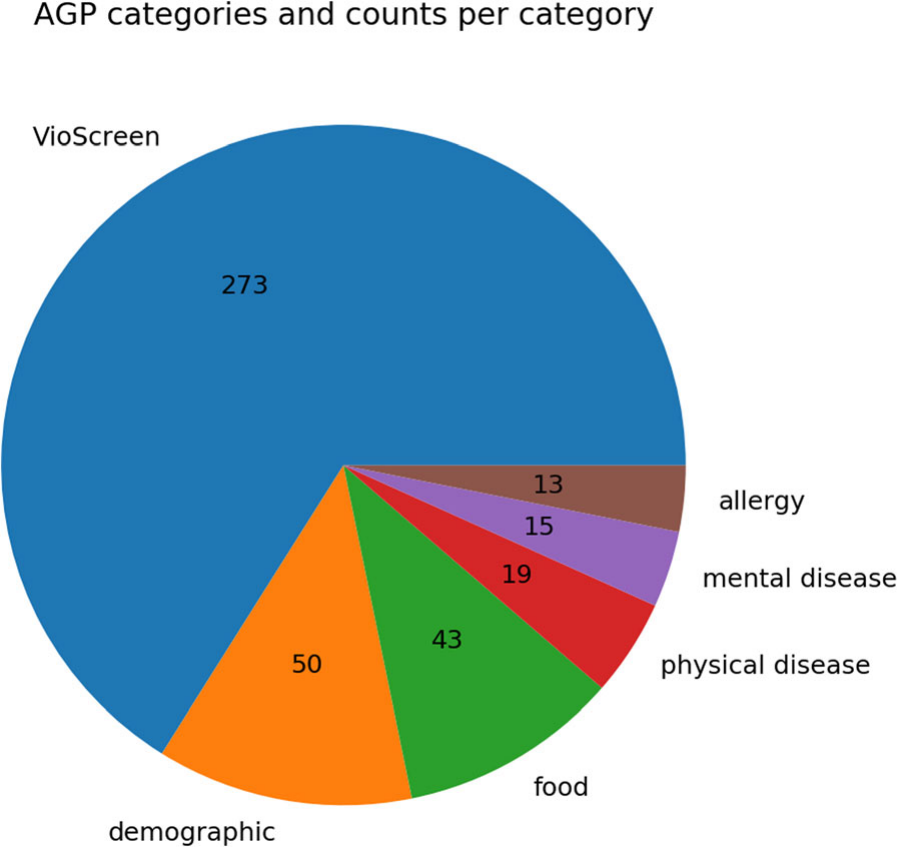
AGP metadata categories

**Fig. 4:**
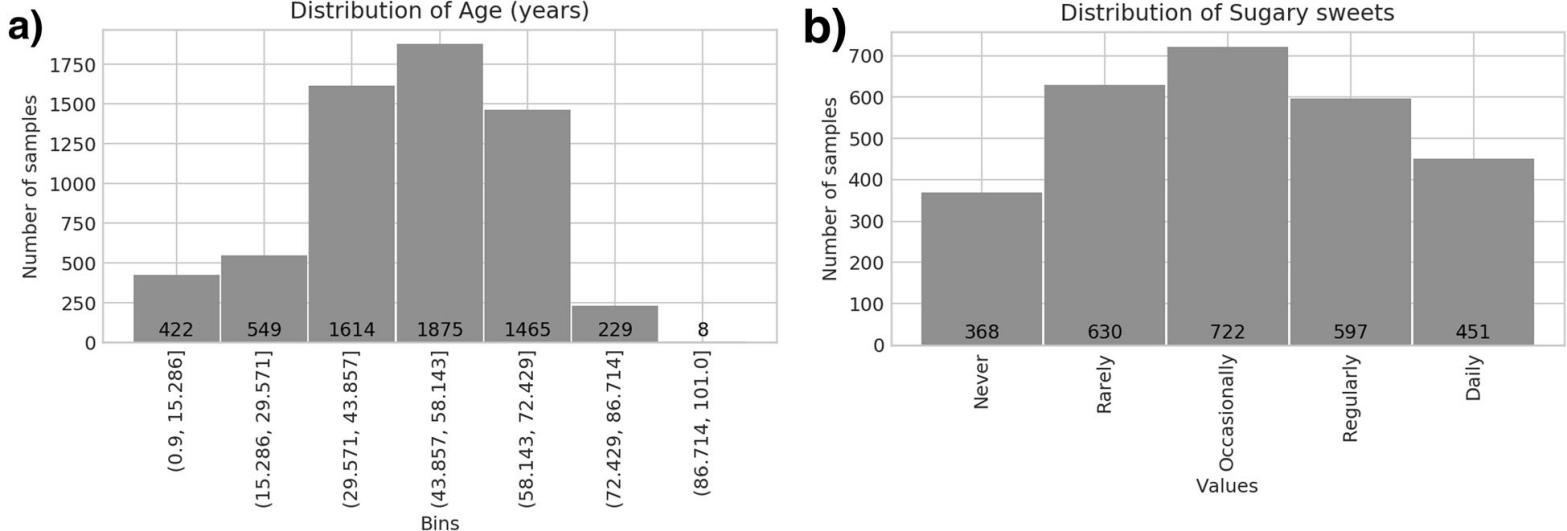
Distributions of selected AGP metadata variables. Both age (**a**) and frequency of sugary sweet consumption (**b**) show a wide range of values enabling re-analysis. For variables involving frequencies of a given activity, such as dietary consumption, data was re-coded in terms of frequency per day (thus, a value of 1.0 in Fig. 4b indicates daily sugary sweet consumption)

EBI’s repository was chosen as the primary source of data for the project. As of today, it is a home to over 1500 public projects, with more than 90,000 samples from various environments, among which the most abundant are human digestive system, soil, rhizosphere, rumen of ruminal animals, water bodies, etc. The API for EBI Metagenomics portal is under development [https://github.com/ProteinsWebTeam/ebi-metagenomics]. The data was acquired from the EBI Metagenomics portal with the aid of the MGPortal data retrieval script [https://github.com/ProteinsWebTeam/ebi-metagenomics]. The sample metadata was downloaded from the American Gut repository [https://github.com/biocore/American-Gut]. The American Gut Project is the main source of data for MNEMONIC. From the project metadata, we extracted 139 variables that could be coerced into numeric data, assigned them to 5 major categories, as well as 273 VioScreen-derived variables, and used them for further modeling of abundance changes (Figs. 3 and 4).

The ProTraits database annotates microbial species with variables that relate to their metabolism, phenotype, or ecosystem. The annotations were obtained by the means of text-mining of the scientific literature, and contain both pre-defined variables and novel variables inferred from the text. The predictions are augmented with comparative genomics, gene pattern similarities, codon usage, proteome composition and co-occurrence in the metagenomic data. The original dataset provides information on the probability that there is a link between the taxon and the trait in question. It contains 3046 species that overlap with the AGP dataset, that can be annotated with 424 variables [60]. In this project, we download the binarized dataset with the cutoff at 0.9 from the ProTraits website [http://protraits.irb.hr].

EBI metagenomics database also provides the functional data, in the form of GO category annotation, paired with the corresponding OTU counts for sample. To evaluate the consistency of the OTU count data obtained from EBI, we compared the results obtained from the OTU matrix and GO matrix in terms of distances between samples that had both annotations. If the results are similar, the matrices obtained with either of the method should also be similar. A Mantel test was performed on affinity matrices calculated from both sources. A correlation of 0.25 on 10,439 samples from various environments was significant (*p* < 0.001), establishing that there is resemblance between the two similarity matrices. More importantly, the permutation determined that the similarity is non-random.

**Fig. 5:**
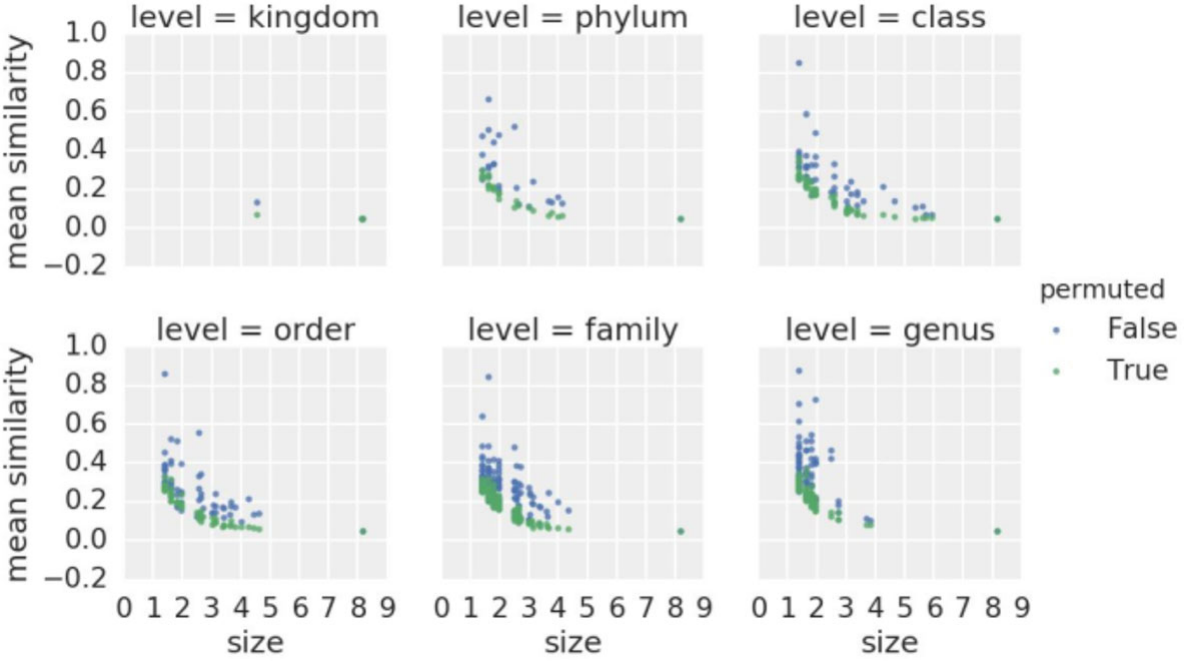
Mean similarity within taxonomic levels. “size” is equal to log(number of species). The transformation of counts has been applied with relation to taxonomic units

Another way to evaluate the data is to compare the similarities between taxonomic groups to a similarities derived from a permuted dataset, based on the OTU count data. Logically, the similarities within a higher-level taxonomic group (e.g. within a genus) should be higher than those of a sample of the same size of random taxa derived from the permuted table (species from mixed genera). We indeed do observe this behavior for all taxonomy level from kingdom to species (Fig. 5).

### Differential abundance matrix

To determine differentially abundant taxa for each of the metadata variables in the AGP’s metadata matrix, we first eliminated samples with fewer than 10,000 mapped 16S reads and taxa with fewer than 1 mapped read across all AGP samples. The primary reason for excluding low-abundance taxa from further analysis was that we are interested in differential abundance *vectors* (i.e., the ordered set of fold-changes for differentially expressed taxa) for particular conditions, rather than individual taxa per se. As mean abundance of an OTU decreases, so too does the magnitude and variance of fold-changes for those OTUs, making it more difficult to stably compare conditions in terms of their overall shifts.

After these filtering steps, the differential abundance matrix was constructed as follows. For each metadata variable in the AGP:

1. Samples with missing values for the metadata variable were dropped.
2. A univariate negative binomial model was fit between the OTU count matrix and the metadata variable using the edgeR package [61]. The model is of the simple form: *Count ~ MetadataVariable*.
3. From the model, the magnitude and significance of each OTU’s univariate association between abundance and that metadata variable were obtained using edgeR’s generalized likelihood ratio test (glmLRT).

After repeating this procedure for each metadata variable, the results are concatenated into a single matrix. The result of this procedure can be conceptualized as a matrix of fold-changes for each OTU and experimental condition. This matrix of measures of differential abundance, or “shifts”, can then be used to determine the similarity between each condition’s abundance changes, or between a query “shift” and the database of AGP differential abundance vectors. Such matrices are fit for each level of the taxonomy (e.g., species, genus, phylum, etc) after collapsing the input abundance matrix to the appropriate level by summing the counts of all child taxa.

## Results

**Fig. 6:**
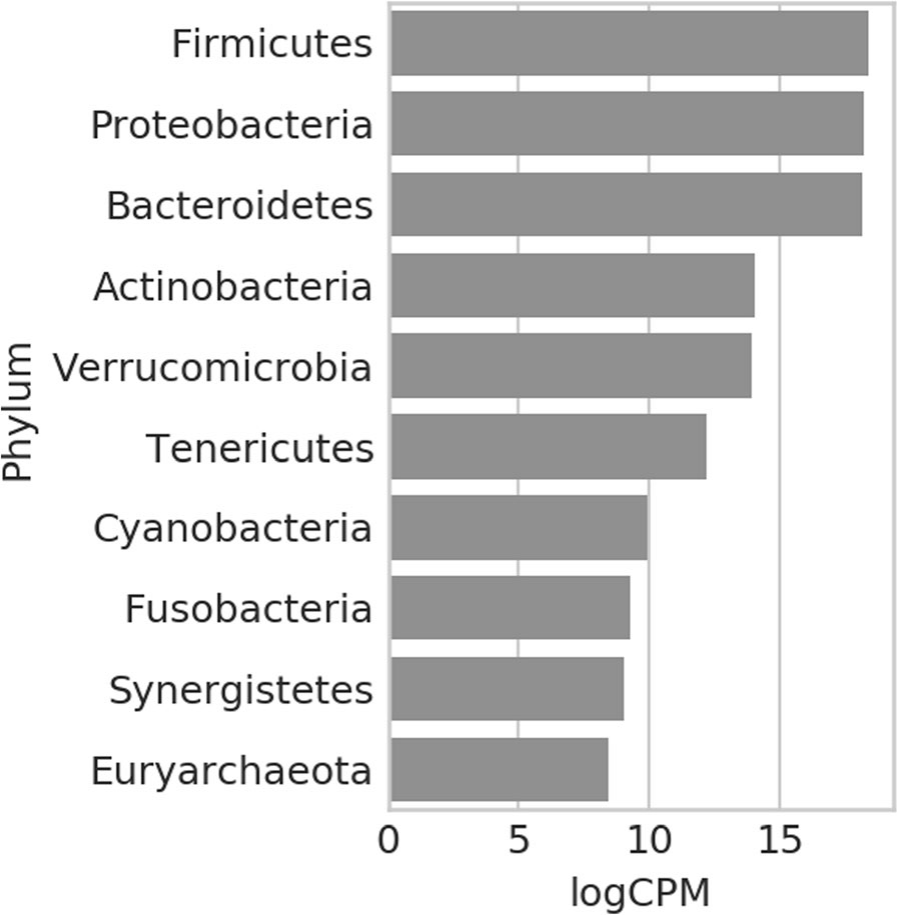
Most abundant phyla in AGP dataset. Globally, within the AGP data, we observe that the most abundant phyla are Firmicutes, Proteobacteria, and Bacteroidetes. This is consistent with previously reported results

**Fig. 7:**
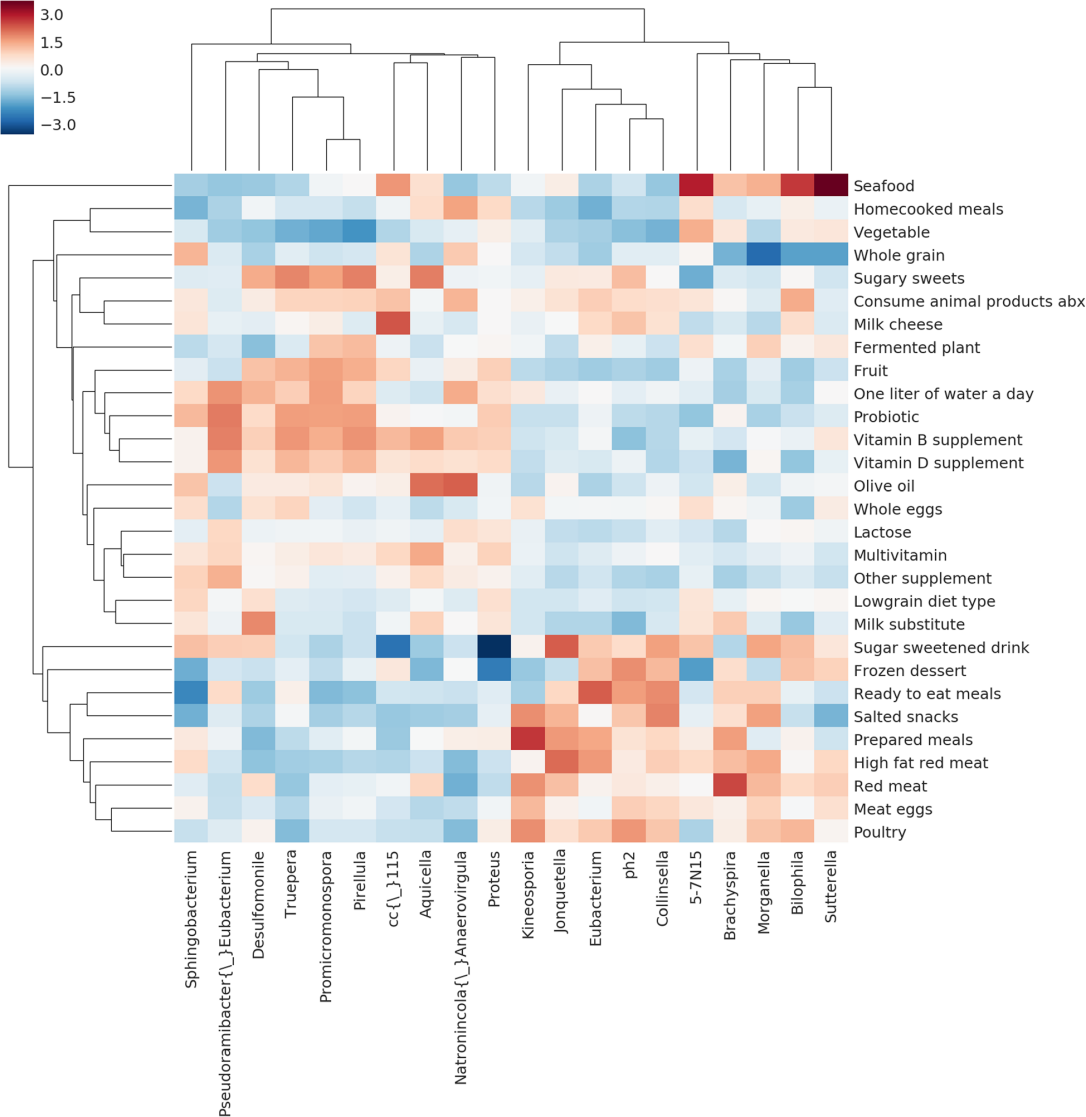
AGP food differential abundance vectors cluster dietary terms by metabolic content. Each value in the heatmap represents the log-fold-change of each species-term combination, normalized across rows by Z-score. Feature selection on genera was performed by identifying the 20 genera most capable of dividing terms into 2 clusters using the ANOVA F-statistic. The resulting clustering of diet-related terms broadly divides dietary differential abundance vectors into protein-rich, simple carbohydrate, and complex carbohydrate clusters

The MNEMONIC package allows users to query and generate analyses of AGP and EBI metagenomic abundance data and differential abundance vectors, as well as compare user-provided data or shifts to public datasets. Figure 6 presents the most abundant phyla in the AGP dataset, among which are Firmicutes, Proteobacteria, and Bacteroidetes. This is consistent with the previously reported findings. Figure 7 shows a clustermap of diet-related terms between food metadata variables in the AGP and 20 differentially abundant microbial taxa within this term set. Microbial abundance associated with food terms results in clusters reflective of dietary considerations. The effect of refined sugar-containing diets on the microbiome has been well-characterized, even after controlled for obesity [62]. Similarly, we see foods with related nutritional content like sugar-related food terms including sugary sweets, frozen dessert, and sugar sweetened drinks cluster closely. Recent studies have shown microbial shifts associated with meat-containing diets and vegetarian diets, reflecting the clustering shown in Fig. 7 [63]. While meat terms like red meat, poultry, and seafood are closely related terms in the dendrogram, their closest related terms like eggs, vegetable and grain suggest food processing, and food terms indicating minimal processing, have a notable effect on microbial abundance. This effect also extends to other foods with limited processing also typically considered part of a healthy diet - vegetable, whole grain, olive oil, and home-cooked meals are closely clustered terms in the dendrogram. This type of change in microbial abundance has been previously shown in dysbiosis resulting from diets containing processed food additives like dietary emulsifiers [64].

Also of note is that probiotics and lactose have similar DA shifts and are grouped somewhat closely, possibly indicating that lactose-annotated samples were capable of lactose metabolism. This effect on microbial abundance is then observed in samples annotated with probiotic use, which is often used to confer lactose metabolizing bacteria in the case of lactose intolerance. This type of alleviation of lactose intolerance has been previously shown in studies like Vonk et al. using probiotic yogurt [65].

**Fig. 8:**
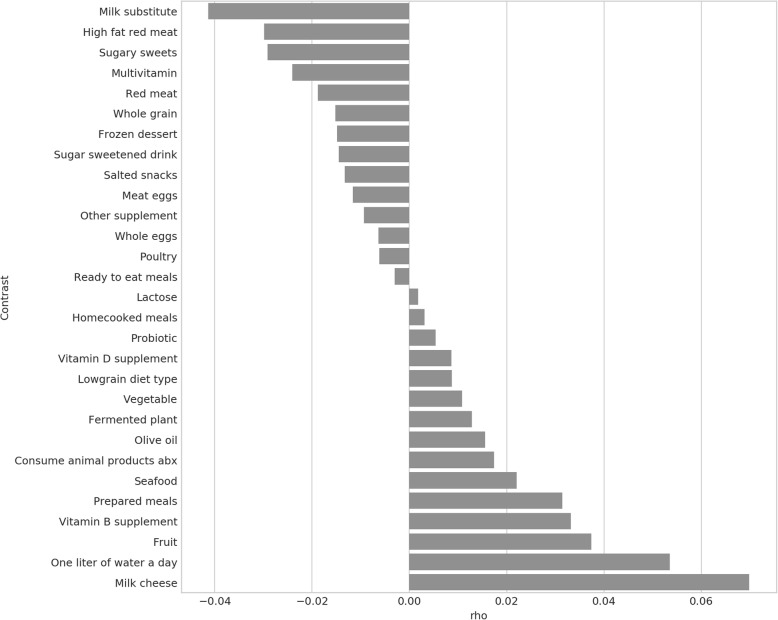
Protein-rich foods are associated with decreased microbial diversity in the AGP. For each AGP sample, the Abundance-covered Coverage Estimator (ACE) metric of microbial alpha diversity was computed using the scikit-bio Python package (https://github.com/biocore/scikit-bio), and the resulting vector compared to each dietary metadata variable using Spearman’s correlation. A high or low rho indicates a respective increase or decrease in microbial diversity is associated with increased frequency of consumption of this type of food within the AGP

Nitrogen-restricted diets are also known to promote healthy aging in mice, possibly via improving microbial community structure [66], and a variety of diseases are correlated in severity or occurrence with microbial diversity; either directly, such as colon cancer [67] and systemic lupus erythematosus [68], or inversely, such as inflammatory bowel disease [69] and rheumatoid arthritis [70]. To assess whether dietary effects were associated with changes in microbial diversity in the AGP, we correlated the ACE metric [71] for microbial diversity with the frequency of consumption of various dietary items. Figure 8 shows that higher consumption of high-protein items is broadly associated with decreased microbial diversity, whereas consumption of probiotics, fruits and vegetables, and milk products is associated with increased diversity. This is consistent with previous findings showing that a high-protein diet decreases microbial diversity compared with a balanced protein/carbohydrate diet [72], fruit, legume, and vegetable-rich “agrarian” diets increase gut microbial diversity [73].

In order to further assess the role of metabolic changes during microbiome-associated diseases, we obtained a matrix of predicted metabolic roles and other traits of microbial species from the ProTraits database [60] and correlated those predicted probabilities with the vector of log_2_ fold changes for AGP differential abundance vectors. We found that dietary fruit consumption was associated with a significant increase in OTUs annotated with fructose metabolism, whereas red meat consumption was significantly associated with increases in microbes annotated with metabolism of amino acids including aspartate and glutamate, as well as alkaline phosphatase and lipase C14 metabolic activity (Additional file 1).

**Fig. 9:**
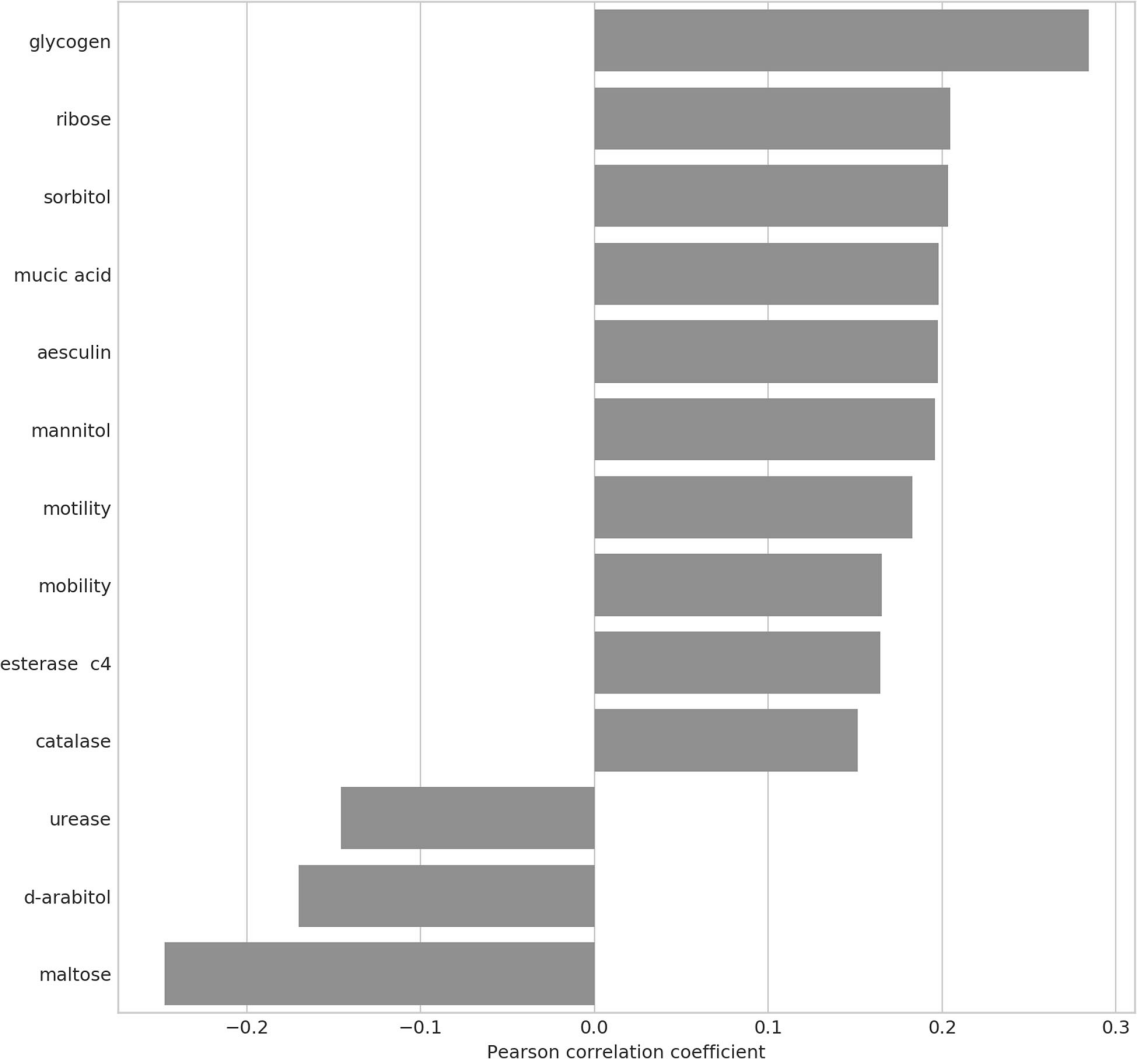
ProTraits metabolic traits associated with diabetes. The differential abundance vector of log2FCs per species in diabetes was correlated with the vector of probabilities for association of each OTU with that metabolic property as provided by the ProTraits classifier. Only significant (*p* < 0.05) associations are shown. A positive correlation coefficient thus indicates an increase in abundance of OTUs predicted or known to metabolize the given metabolite

**Fig. 10:**
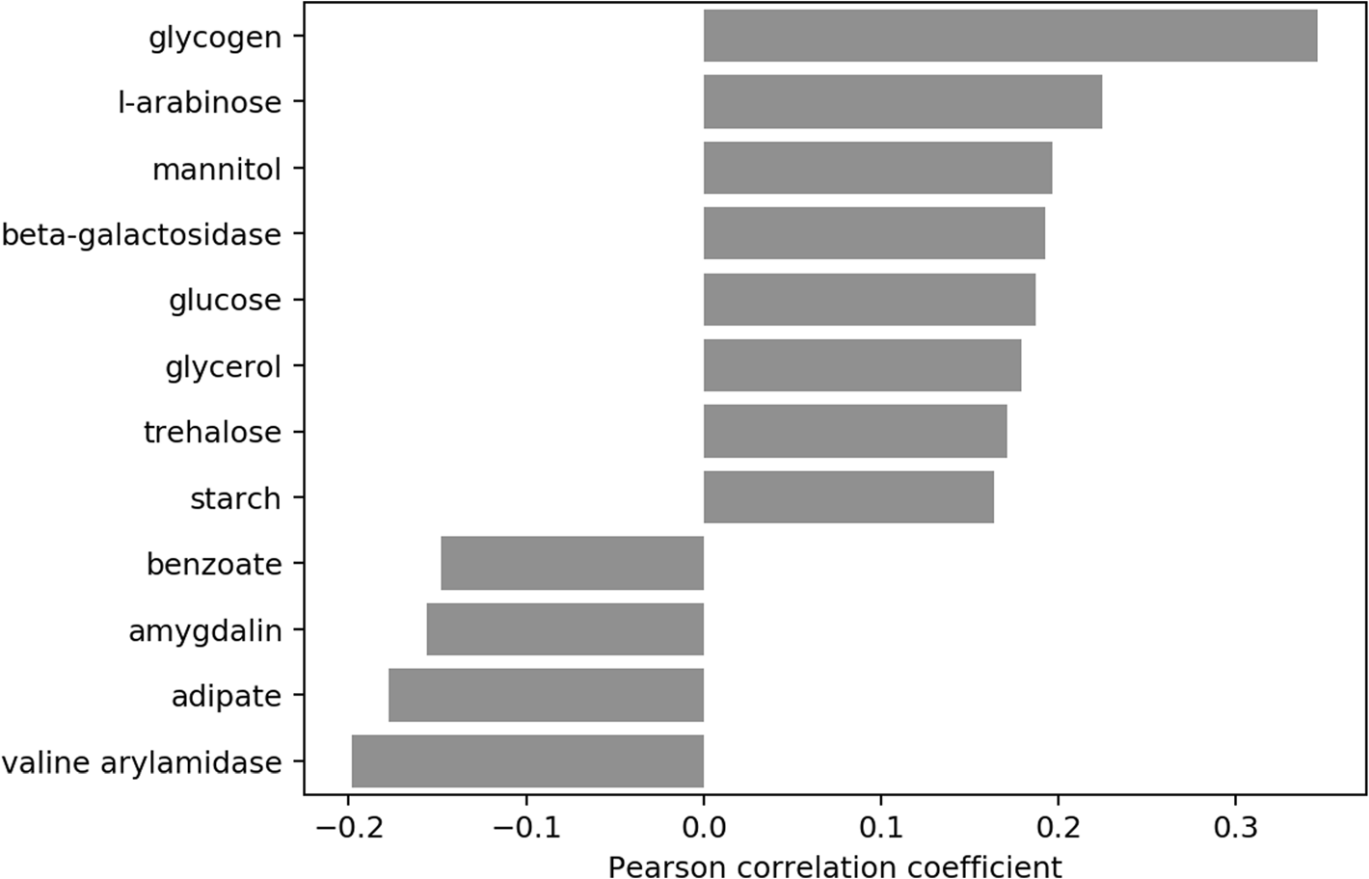
ProTraits metabolic traits associated with IBD. The differential abundance vector of log2FCs per species in IBD was correlated with the vector of probabilities for association of each OTU with that metabolic property as provided by the ProTraits classifier. Only significant (*p* < 0.05) associations are shown. A positive correlation coefficient thus indicates an increase in abundance of OTUs predicted or known to metabolize the given metabolite

We then applied this analysis to diabetes mellitus (DM; Fig. 9) and inflammatory bowel disease (IBD; Fig. 10), and found that whereas both were associated with significant increases in glycogen-metabolizing microbes, DM was also marked by an increase in metabolism of the monosaccharide ribose and disaccharide cellobiose, as well as the sorbitol, which is often used as a sugar substitute, and a decrease in maltose metabolism. Decreasing maltose metabolism has been reported in literature to improve blood glucose after sugar challenge and reduction in maltase activity is a desired effect in anti-diabetic therapies such as trigonelline [74, 75]IBD was associated with increases in starch and beta-galactosidase activity. Broadly, then, both disorders are marked by a shift of the microbiome towards carbohydrate metabolism.

**Fig. 11:**
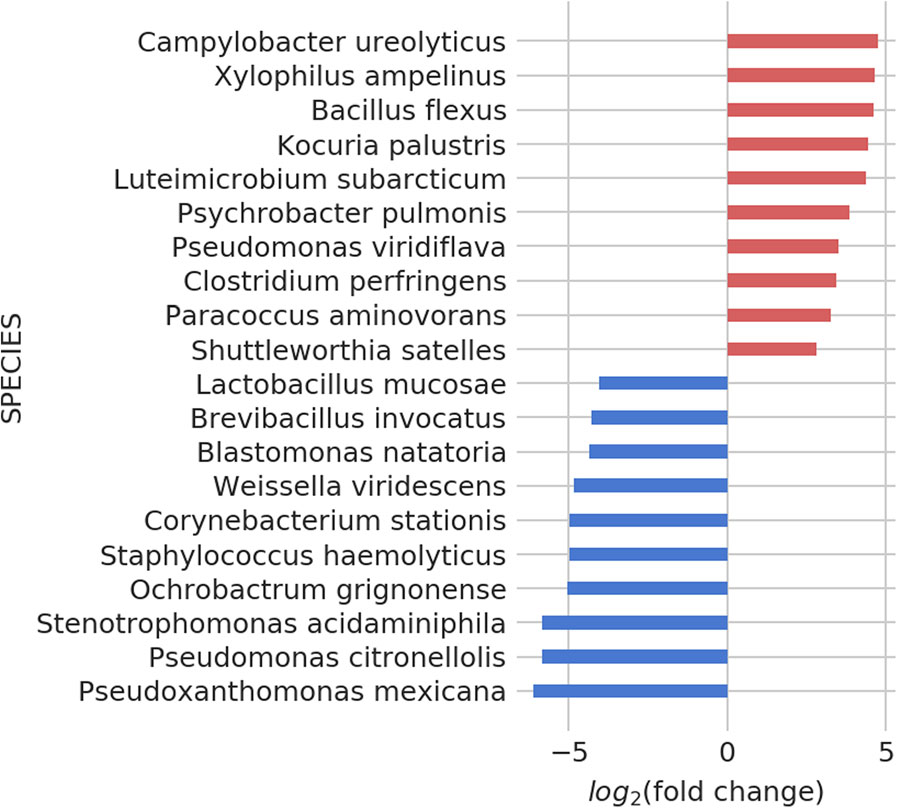
Changes in microbial species in autism spectrum disorder patients

There is increasing amount of epidemiological evidence that the microbiome might be involved in the development of late-onset autism. Antimicrobial therapy seems to precede the symptoms [76] and a subsequent vancomycin therapy can alleviate the symptoms short-term [77]. It has been proposed that some gut microbes may produce neurotoxins that would make the autism symptoms worse [78]. Many autism patients also exhibit GI tract problems during the onset of the disease which often persisting [79, 80]. In AGP samples, we observe an increase in some species associated with poor water quality (*Bacillus flexus, Kocuria palustris*), and food poisoning (*Campylobacter uroelyticus*, *Clostridium perfringens*) (Fig. 11) [81]. *Campylobacter ureolyticus* has been shown to be increased in Crohn’s disease and other GI symptoms [82]. Autistic children have been reported to have elevated levels of ammonia in stool [83], a compound which *K. palustris* can degrade well [patent no CN103103141-A]. *Kocuria* species have been reported to be contributing to brain abscess and meningitis, as well as a cause of urinary tract infections [84, 85]. Some of the bacteria that showed up are seemingly unrelated to the condition, like *Xylophilus ampelinus* (a plant pathogen). Most of the bacteria decreased in ASD are non-pathological, environmental species: *Pseudoxanthomonas mexicana*, *Pseudomonas citronellolis, Blastomonas natatoria*, with the exception of *Staphylococcus haemolyticus*, which is a known hospital pathogen [86].

## Discussion

The widespread availability of public metagenomic data enables new data to be interpreted within the context of prior experiments. As more data is collected, more and more accurate statements can be formulated about the interactions within the microbiome, as well as between the microbes and the environment. As of today however, despite the fact that there are many samples available publicly spanning different environments, the data may prove somewhat selective. Some conditions are represented by a single project, some lack a ‘control’, or a suitable comparison; some are just lacking the sample annotation needed to draw any conclusions.

Moreover, there are a plethora of confounding factors which cannot always be controlled for. For example, human subjects find it difficult to stick to a strict diet, which introduces higher variability in the dietary input, which in turn changes the microbiome in a more convoluted way. We also have highly variable - individually and between each other - behavioral patterns. We differ genetically and with respect to background, which have also been shown to affect the gut microbiome. Finally, most of the variables - notably diet-related - are self-reported, which makes the data prone to a bias introduced by many observers, as well as simple forgetfulness.

All of the factors mentioned above limit the applicability of the method we propose. That said, the American Gut Project is a source of highly standardized information about the samples, and there is a decent number of samples in it. We also expect the trend in data accumulation to stay increasing. In a few years, there will be more data available, and this will widen the bottleneck we’re faced with currently.

Being still a relatively young field, metagenomics suffers from the lack of standardized methods for data analysis, as is the case with e.g. gene expression analysis. The EBI analysis pipeline is an example of a proposed standardized solution for metagenomics data analysis up to converting the raw data to counts of different taxa or genes. Using such a pipeline allows us to minimize the bias introduced by technical variability. The choice of methods for downstream analysis is less obvious. There have been efforts undertaken to compare different computational tools to each other and evaluate the performance of any individual method for differential abundance calculation, clustering, etc. The modeling strategy we used in MNEMONIC seems to be performing well, but may not be optimal. One caveat of using gene expression analysis tools for metagenomics data is that, because of the sequencing technology limitations, the latter is compositional and does not represent absolute counts, which in turn implies that data is not independent [87, 88]. This characteristic will cause many standard statistical tools to yield false positive results. The data is also more sparse than the RNA-seq counts, which further complicates modeling [89]. However, despite difficulties associated with the analysis of metagenomics data, studies consistently show that some signal can be achieved even using simple statistical methods, and is strong enough to overcome the technical bias [41, 90].

In addition to these issues, without a gold standard, we cannot quantitatively evaluate the performance of the approach and are relegated mostly to “sanity checks” (i.e., replicating findings well-established by others). Lastly, when studying metagenomics in the context of human gut, it is not clear, in most cases, whether the observed or potential changes in microbiome are the cause of a pathology, the effect, or just accidental. It could also be the case that they contribute both to the cause and the effect, with potential complex interactions and feedback loops, and the interplay of host condition and microbiota composition converges, over time, to a recognizable disease state. For example,a patient with rheumatoid arthritis might go grocery shopping less frequently because of the pain it causes them to walk to the grocery store, resulting in a less diverse or otherwise changed diet and, in turn, changes in microbiome. And perhaps the microbiome changes, in turn, exacerbate their health issues, resulting in a cycle of less frequent shopping and worsened condition.

One should be also wary when interpreting results from a metagenomics experiment in a certain context. As an example, dysbiosis is widely defined as a generic disturbance of the “correct” microbiome [91] and seems to be associated with a variety of diseases. Interestingly, different diseases seem to have a similar pattern of change with respect to the ‘healthy’ microbiome [90]. However, it’s been suggested that the host-microbiome interactions may be much more complex and that the term is not only overly general, but also misleading [92]. Even with so many factors influencing the microbial composition, it may be possible to define a ‘healthy’ human gut microbiome or a ‘core’ gut microbiome, but necessarily with tolerance to the relatively high group or individual variation.

## Conclusion

We present an approach to help interpret new metagenomics experiments, specifically as an algorithmic means of searching for similar “shifts” that have been reported within public metagenomic repositories in terms of differential microbial abundance between conditions. The question itself is important to establish whether a newly observed change in the microbiome has been seen before and, if so, under what conditions. This is important in interpreting new results, as a merely descriptive report of changes in microbial fractions does not answer the question about what that change might mean in terms of its potential relevance to the host. If our observations of microbial abundance shifts are similar to others, then the nature of their experiments informs us as to how to interpret ours. For example, examining the experiments that led to similar microbiome changes might yield a unifying theme such as changes in dietary composition (salt, protein, fat, sugar, etc), immune activation/repression, or response to stress. In turn, if we are studying diseases, then similar shifts might lend themselves to testable hypotheses regarding causality. Alternatively, if no previous experiments are highly similar, then knowing this enables us to claim our observations are novel.

The main limitation of this report is that we cannot yet automatically (algorithmically) detect from the meta-data alone which samples within an experiment are control and which ones experimental. In some cases, there may be only controls (e.g., a survey of microbial abudance), or there may be multiple comparisons that involve either different experimental perturbations or multiple time-points for one perturbation. The AGP enabled us to bypass this limitation for now by focusing on one that came with well-annotated structure. In the future, for the potential for this approach to be fully realized, the reporting of experimental vs control conditions needs to be easy to recognize algorithmically. One solution would be to require increased structure to the meta-data reporting in new microbial experiments, but another would also be to increase our ability to algorithmically extract the necessary values from a free-form description either in the meta-data or publication itself.

## Additional file

Additional file 1: Association of fruit and meat consumption with metabolic variables. Dietary fruit consumption is associated with a significant increase in OTUs annotated with fructose metabolism, whereas red meat consumption was significantly associated with increases in microbes annotated with metabolism of amino acids, alkaline phosphatase, and lipase C14 metabolic activity. (PNG 70 kb)

## Abbreviations

AGP: American Gut Project
DA: Differential abundance
EBI: European Bioinformatics Institute
