# Supplementary figures and images for: MNEMONIC: MetageNomic Experiment Mining to create an OTU Network of Inhabitant Correlations
Source: BMC Bioinformatics. 2019 Mar 14;20(Suppl 2):96. doi: 10.1186/s12859-019-2623-x (PMC6419333; doi:10.1186/s12859-019-2623-x)

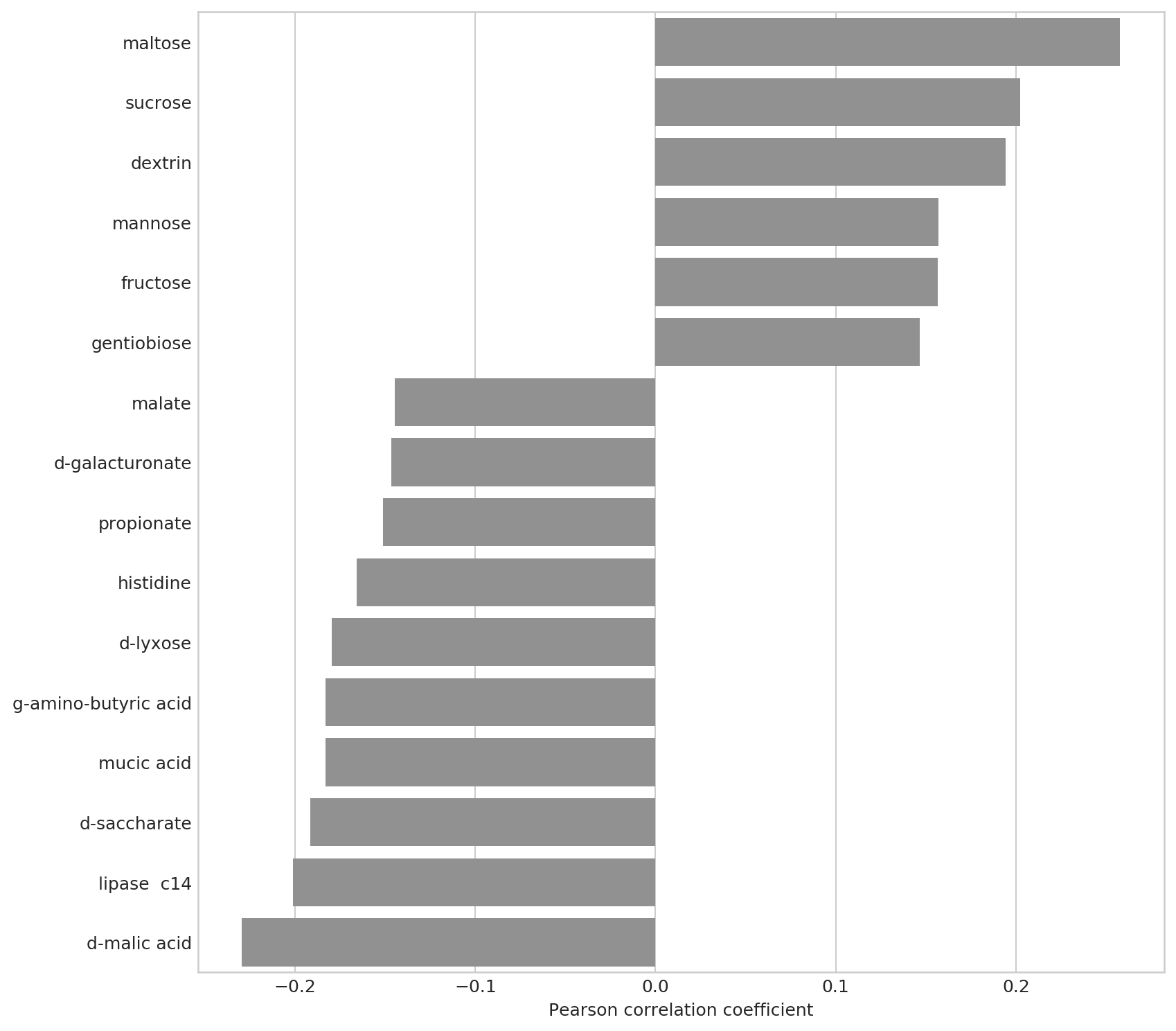

Supplement: Supplementary file 1 — Association of fruit and meat consumption with metabolic variables. Dietary fruit consumption is associated with a significant increase in OTUs annotated with fructose metabolism, whereas red meat consumption was significantly associated with increases in microbes annotated with metabolism of amino acids, alkaline phosphatase, and lipase C14 metabolic activity. (PNG 70 kb) [file 12859_2019_2623_MOESM1_ESM.png]
